# Social facilitation of trotting: Can horses perceive and adapt to the movement of another horse?

**DOI:** 10.1371/journal.pone.0309474

**Published:** 2024-08-26

**Authors:** Paulo Moreira Bogossian, Juliana Santos Pereira, Nathalia Felicio da Silva, Ayrton Rodrigo Hilgert, Sarah Raphaela Torquato Seidel, Joice Fülber, Carla Bargi Belli, Wilson Roberto Fernandes

**Affiliations:** Department of Internal Medicine, University of Sao Paulo, School of Veterinary Medicine and Animal Science, São Paulo, Brazil; Yuan Ze University, TAIWAN

## Abstract

Exercise intensity is prone to be self-regulated in horses exercising freely. The main drivers include social, feeding and escape behaviors, as well as the operant conditioning. We hypothesized that self-regulated exercise intensity may increase due to the presence of another horse exercising ahead. Seven horses were assigned to a 2x2 crossover trial following treadmill familiarization. Video images of a trotting horse were displayed on the wall in front of the experimental unit (Visual), which was positioned in the treadmill. Physiological and behavioral markers were further compared with a control visual stimulus (Co), comprising a racetrack image without horses. Horses were sampled during a constant load exercise test (1) at rest (baseline), (2) after the warm-up (0 – 10^th^ minute) and (3) after visual stimulation or control (10^th^– 12^th^ minutes of the SET) to quantify plasma lactate and glucose concentration, heart rate, head angle, as well as behavioral markers. Following visual stimulation, heart rate (130.8 ± 27.8 b.p.m.) was higher than control (84.7 ± 15.1 b.p.m., P = .017), as was plasma lactate (Visual ‐ 5.28 ± 1.48 mg/dl; Co -3.27 ± 1.24 mg/dl, P = .042) and head angle (Visual ‐ 36.43 ± 3.69°; Co -25.14 ± 4.88°, P = .003). The prevalence of “ears forward” behavior was also higher following Visual (100% - 7/7) than Co (14% - 1/7, P = .004). These results suggest that visual stimulus (1) was safe and well tolerated and (2) prompted the anaerobic lactic pathways and shifted the behavior to a vigilant state. In conclusion, horses were able to perceive and adapt to a social environment. Our findings validate the use of social facilitation of trotting to encourage horses to move forward avoiding the use of the whip.

## Introduction

Exercise intensity, defined as the gradient of neurohumoral and metabolic response to physical activity [1], can be determined from blood lactate levels [2]. Various factors that increase hind and forelimb workloads, such as speed and inclination [3], as well as the ground surfaces [4] are prone to increase exercise intensity. In addition, exercise intensity may increase voluntarily due to feeding, breeding, and climate [5,6], or non-voluntarily, due to human’s action including the use of the whip or leg pressure. In the past 20 years, welfare concerns surrounding practice in equestrian sports, such as cervical hyperflexion [7] and the use of the whip [8], have been scrutinized. Nevertheless, ethical, non-violent approaches helping horses to increase the intensity of the movements voluntarily have been overlooked, nor has the animal’s capacity to perceive and to adapt to the stimulus been studied.

Social facilitation of trotting, defined as an increase in response merely from the sight or sound of others making the same movement [9], has been widely adopted by endurance and flat-racing trainers to (1) to encourage those horses who need extra stimulus to move forward and (2) to calm excited horses [10]. Although social facilitation of movement has shown to be a promising and well accepted training practice in racehorses [11], there is limited evidence demonstrating its effect on horse behavior and physiology. Indeed, equitation is known to suppress individual horse voluntary actions, as there is a novel and strong signaling path being established by the rider [12]. Because modern horses still display many adaptive behaviors, the natural urge to follow or to be followed remain even in well trained horses and may be a cause of stress in solo rides. On the other hand, subtle and voluntary increase in hindlimb workload in response to the presence of another horse moving ahead may favors racing performance, therefore deemed to be a worthy trait [11].

The behavior of following another horse is essential to maintain group cohesion during collective departures in feral horses [13]. A recent survey demonstrated that propensity to lead or follow another horse in response to group departure differs among horses within the same group [14]. It shows that personality and interactions may shape the organization of collective movements. Briard et al. (2015) found that the bolder the individual were, the more the start attempts they made [14]. At the individual level, the ability to control the movements demands the central nervous system (1) to monitor the whole-body movement, wherein vision and inner ear organ semicircular canals are required to provide information about location and orientation and (2) neuromuscular activation, aiming to compensate, adjust or initiate body movements [15]. Sensory information is likely transformed into appropriate motor action through a complex, well-studied phenomena called sensory-motor transformation [16], which has been essentially related to escape and feeding behaviors [17,18].

Because it is the only inter-species sport included in high level Olympian competitions, equestrian sports represent a complex, multidisciplinary challenge for ethicists, psychologist, and physiologist [19]. Despite the recent advance in Sport horses welfare [20], and the growing public concern around the use of the whip [8,21], there is limited research on using sensory, non-violent stimulus to increase the exercise intensity in horses. The understanding of this sort of stimulus, may prompt the development of novel and ethical training technique for sport horses.

We hypothesized that horses are able to perceive the movement of another horse projected ahead and will increase the intensity of their movements in attempt to follow or overtake it. Also, we hypothesize that social facilitation of trotting may increase horse’s visual and auditory attention, shifting their facial expression.

## Materials and methods

### Experimental design

We designed a randomized repeated measures crossover trial in order to verify the null hypothesis that physiological and behavioral markers of horses undergoing a standardized exercise test with visual stimulus for movement does not differ from a control stimulus. Horses were randomly assigned to one of the following experimental sequences: (1) control-visual: control stimulus followed visual stimulus intervention, CV sequence and (2) visual-control: visual stimulus intervention followed by control, VC sequence. The washout period was defined to be longer than 6 days.

**Fig 1:**
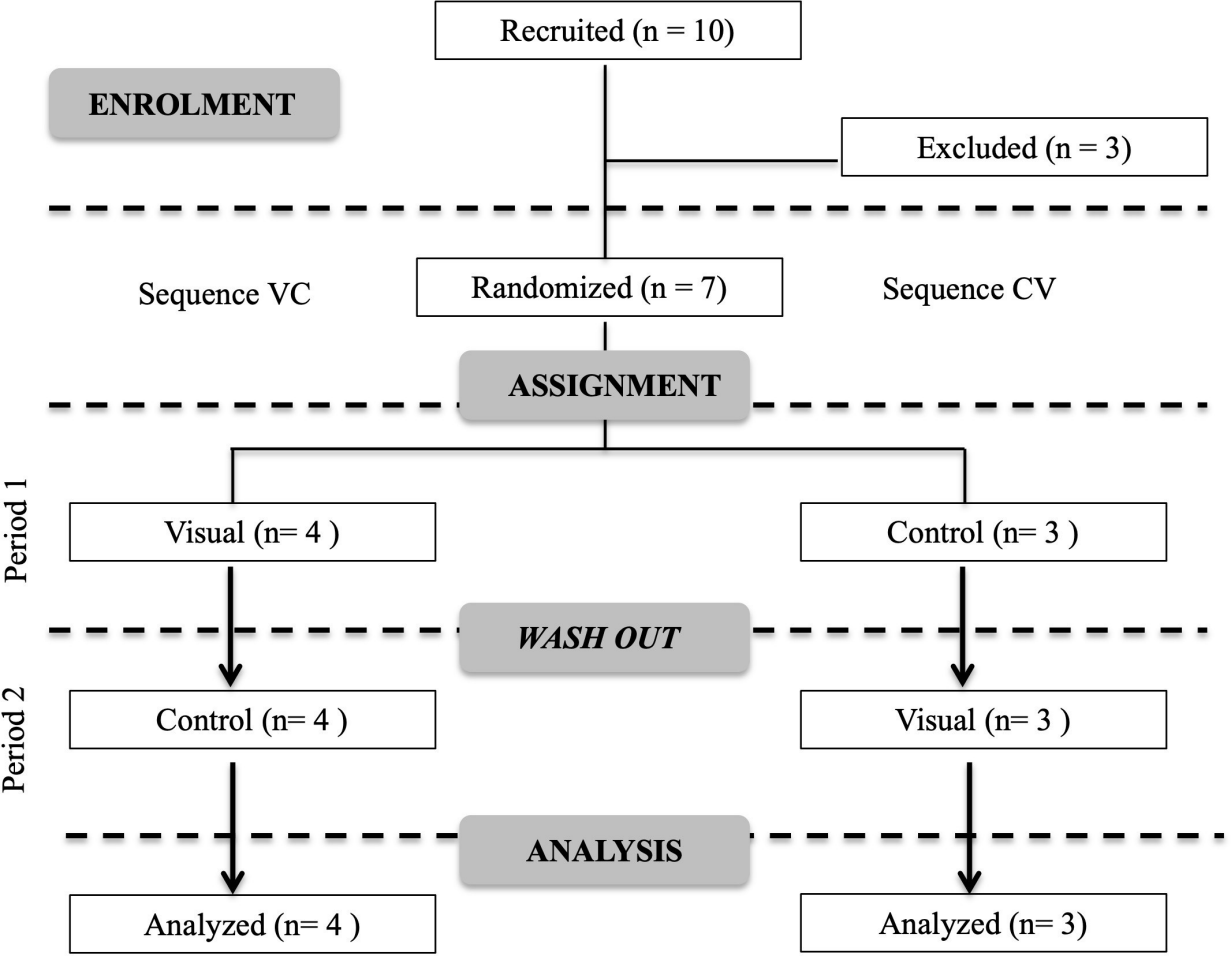
Consort flow diagram, demonstrating the enrollment and assignment processes. Note that ten horses were initially recruited, and three horses were excluded from the trial leaving seven to be randomized. Four horses were initially exposed to control stimuli, then were exposed to visual stimuli (control-visual sequence) while the other three horses were initially exposed to visual stimuli, followed by control stimuli (visual-control sequence).

A convenience sample of ten horses was firstly recruited to this survey, as they matched the following inclusion criteria: no observed abnormalities during clinical examination and calm temperament. Three horses were excluded due to (1) owner request with no formal justification, (2) hoof infectious disease diagnosed during the familiarization period (Fig 1).

### Animals and ethics

The present study was approved by the Ethics Committee on the Use of Animals, Faculty of Veterinary Medicine and Animal Science, University of São Paulo, under protocol number 1216230518.

Seven horses (4 mares and 3 geldings) belonging to the following breeds, Purebred Lusitano (4/7), mixed-bred (1/7) and Arabian (2/7), ageing 4 ± 1 years and weighing of 431.4 ± 86.3 kg were recruited to this survey. All horses were housed in individual boxes (3mx3mx3m) at the Veterinary Hospital of the Faculty of Veterinary Medicine and Animal Science of the University of Sao Paulo, where they received *ad libitum* water, Coast Cross hay and commercial concentrate (1.5% of body weight). The mares did not engage track or treadmill training before the onset of the experiment, being entirely at rest before the familiarization step. The athletic history of the mixed-bred gelding is unknown, but it was entirely at rest for at least 60 days before the onset of the experiment. The Arabian geldings had engaged in treadmill training for one year before the onset of the experiment, but no other riding or driving routine was reported. All horses were daily managed, inspected, cleaned, and habituated to the treadmill room and associated equipment as well as to the treadmill locomotion itself.

### Familiarization period and standardized exercise test protocol

All horses were familiarized with treadmill room and equipment, treadmill locomotion, and projector light for 12 consecutive weeks, comprising up to 3 working sessions per week. Familiarization session comprised 20 minutes of walk and stop around the treadmill (first week), followed by specific reinforcement learning on the treadmill locomotion (2^th^ to 12^th^ week). After 12 weeks of training, no significant increase in heart rate (HR) and no avoidance behaviors were observed when horses walked and trotted on the treadmill surrounded by our research team and with all equipment turned on. Brief familiarization (3 days) to the video images was performed, to simultaneously prevent avoidance behavior and excessive habituation to the video images.

The standardized exercise test (SET) involved the following protocol: (1) warm-up, encompassing five minutes at walk (1.8 m/s), followed by 5 minutes at a trot at 3.2 m/s and (2) visual stimulation (Visual) or control stimulus (Co), comprising two minutes trotting at 3.2 m/s, followed by a complete stop.

### Procedures, sample collection and analysis

**Fig 2:**
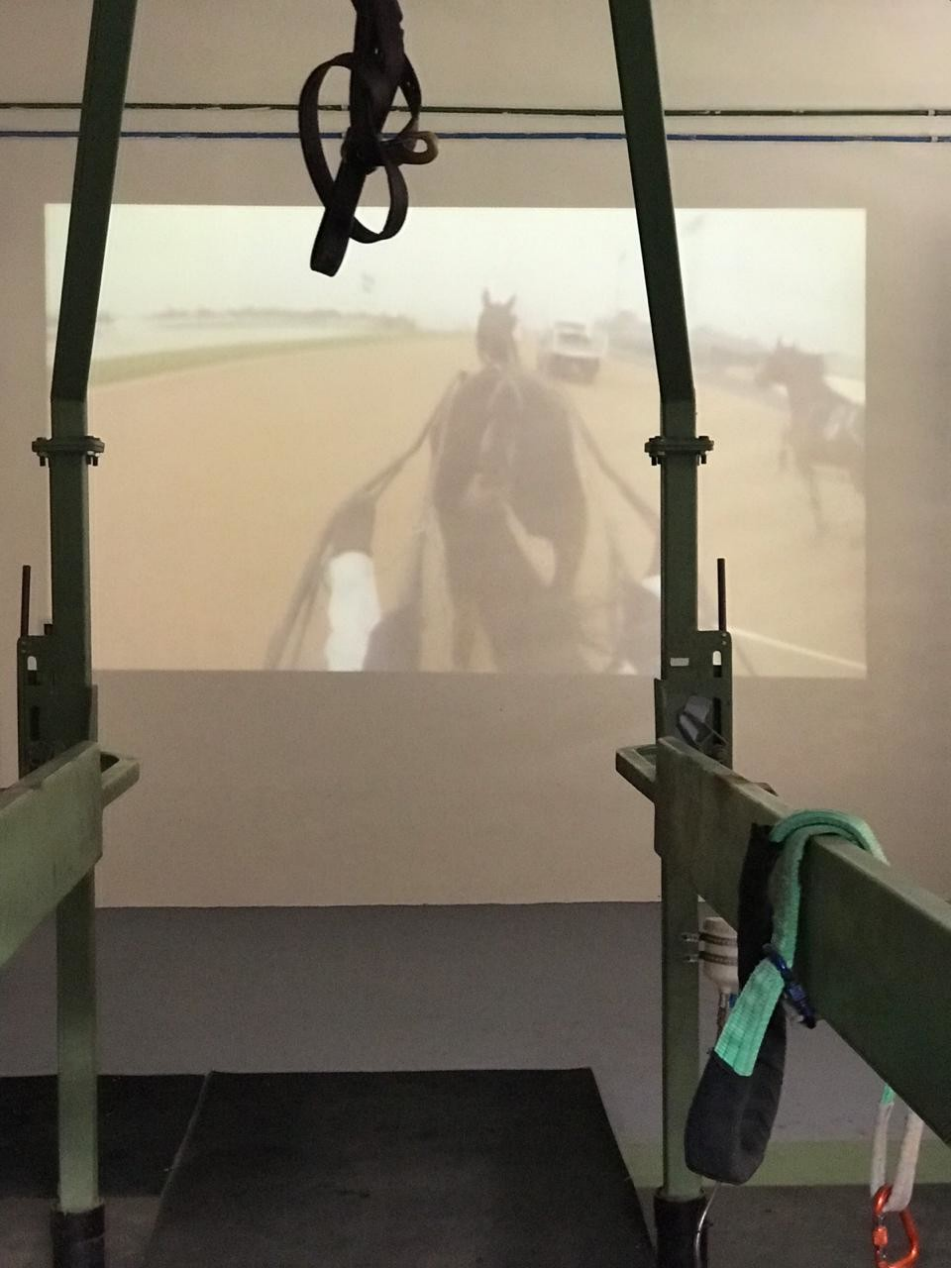
Treadmill view of the video images projected on the wall to promote social facilitation of trotting. Note that horses’ perspective of the other horse moving in front of them is caudal and oblique.

Firstly, we prepared a video (mp4. fie) through recording a Standardbred horse trotting on the track at high speed for two minutes. Images were captured from behind, with the camera positioned on the driver’s head and focusing horse’s head, neck, trunk and proximal hindlimb from an oblique, caudal perspective (Fig 2).

**Fig 3:**
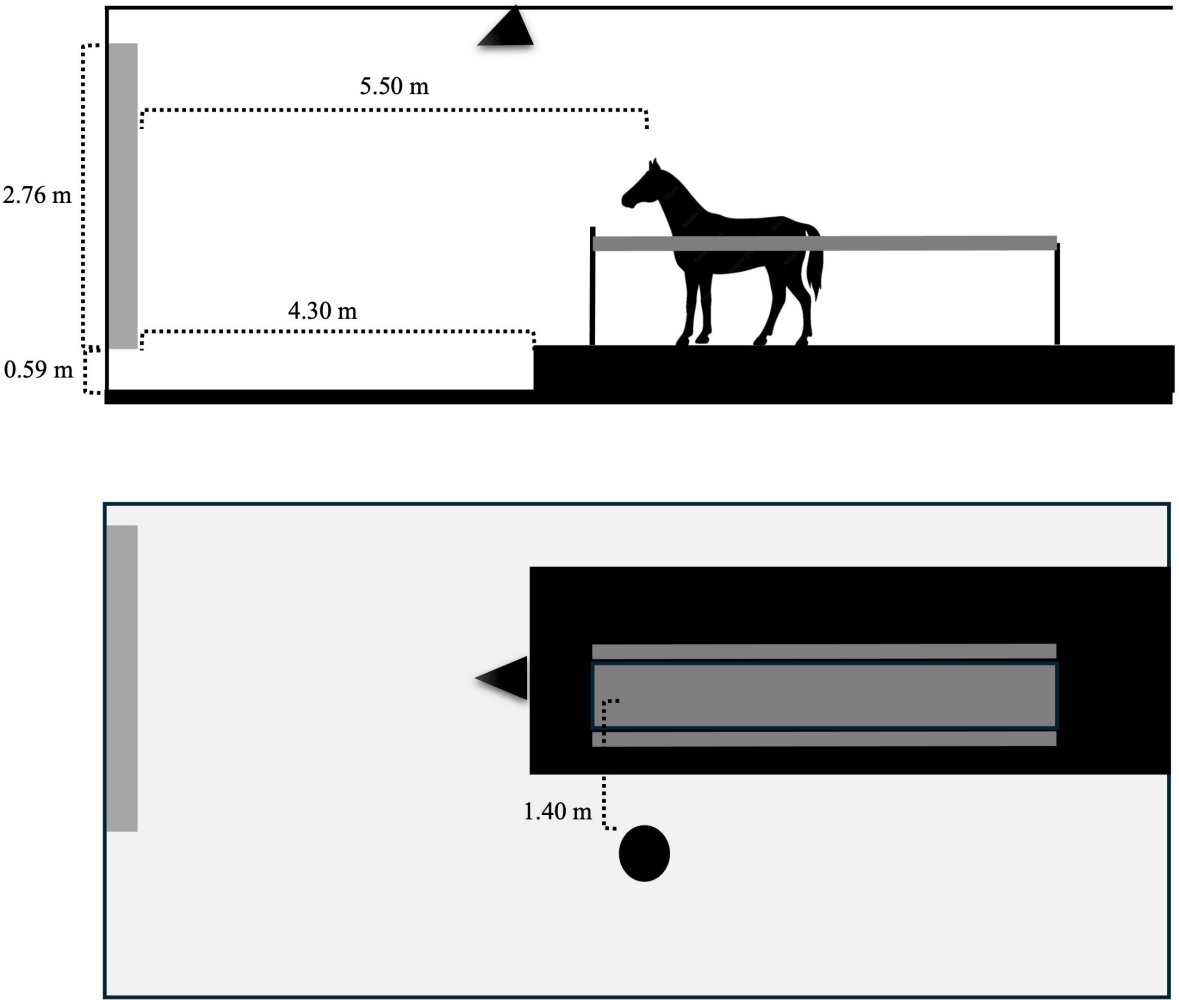
Schematic representation of the treadmill room, including the full setup arranged to assess the effect of social facilitation of trotting on exercise intensity and behavior of horses undergoing standardized exercise test on a treadmill. Note the relative position of the plain white wall where the images were projected on (black solid line on the left), the area of projection (grey rectangle on the left), the treadmill (black solid rectangle under the horse), camera (solid black circle) and the projector (solid black triangle).

This video was projected on a white plan wall, through an image projector (Sony©) connected with a notebook, to promote social facilitation of trotting. Once edited to suppress the emission of sound, we projected the video on the wall 4,3 m away from the treadmill. The height of the image relative to the ground and the distance from the horse’s head were 0.59 m and 5.50 m, respectively. Yet, the dimensions of the image were 3.64 in length and 2.76 in height, corresponding to 1.35 times the horses’ expected average height (Fig 3).

To serve as a control stimulus, we projected a picture of a track, with sand covering the ground and white bars indicating the lateral limits of the track (see S1 Fig). Both track picture (control) and video images of another horse exercising ahead (visual) were projected following the warm-up period. Therefore, only the projector blue light was displayed on the wall in front of the experimental animal during the warm-up period.

Heart rate (HR) measurements were performed at rest and during “warm-up” and “visual stimulus / control” periods. The Polar RS800 heart monitor (POLAR ELECTRO©, Switzerland) was used to continuously record heart rate measurements. Raw data drawn from the first to the 10^th^ minute and from the 10^th^ to 12^th^ minute of the SET were used to calculate the mean heart rate of the warm-up and intervention steps, respectively. Blood samples from the external jugular veins were obtained aseptically and placed in tubes with sodium fluoride for biochemical determination of plasma glucose and lactate levels. An 18G catheter was positioned in the external jugular vein on the left side, and a catheter extensor was attached to it, to facilitate blood aspiration during exercise. Blood aspirations were performed at rest, after warm up following the intervention (Co or Visual).

The samples packed in sodium fluoride vials (Hemogard©, 4 mL) were refrigerated at 6°C until the end of the SET, then centrifuged at 1300g for 10 minutes, and the plasma was aliquoted into microtubes of 1.5 mL (duplicates), and then frozen at -80°C for 14 days. The samples were analyzed using the automatic biochemical analyzer (LABTEST©, MODEL LABMAX 240).

The angle of the longitudinal axis of the head was determined from video images generated by a smartphone (IOS system, USA), positioned on a tripod 1.40 m from the treadmill. Image processing was performed using ObjectVideo 1.6.1© software (Objectus Technology, LLC, USA), which has three important features: slow motion image mode; axis tool, and angle tool. The slow-motion view mode allowed the choice of the frame with greater parallelism, avoiding parallax errors. The axis tool allowed the identification of the vertical axis ‐ straight line with an angle of 90° in relation to the ground ‐ intercepting the nasal plane of the animal. Finally, the angle tool was used, whose vertex was positioned on the nasal region, one of the axes was traced on the previously delimited vertical axis and the other, on the contour of the animal’s forehead towards the nuchal crest. Therefore, the angle measured represents the relative position of the head (nasal plane) in relation to a vertical line. For example, if the horse has positioned its nasal plane exactly on the vertical (“on the bit”) the resulting angle measurement should be zero. If the head was elevated and the neck extended, i.e. “above the bit” or “nose up”, an increased angle were recorded.

The camera positioned 1.40 m away from the treadmill was also used to collect video images of the horse’s body and head for behavioral analysis (Fig 3). The determination of the behavioral classes was carried out from the descriptive inventory of the behaviors exhibited during physical activity on the treadmill, based on the recorded video images. These behaviors were registered in the order they appeared in the videos and described in detail (Table 1).

**Table 1:** Inventory of the behaviors presented by seven horses engaging a constant load exercise test in a treadmill with visual stimuli for movement (Visual) and control (Co).

Behavior	Description	Animals (n)	Repetition (n)
Back off	Move backwards (<1m)	7	23
Head down	Extend the neck and lower the head	3	4
Mouth opened	Separated lips	4	9
Head shaking	Shake the head sideways	3	7
Snorting	Rapid exhalation with nostril vibration	7	44
Nostrils opened	Repeated opening movements of the nostrils	3	6
Head up	Raise the head	4	5
Ears backward	Ears backward (> 5 seconds)	3	6
Ears forward	Ears forward (> 5 seconds)	7	13
Chewing	Chewing movement	2	2
Side looking	Lateral displacement of the head	5	21
Back off II	Move backwards (>1 m)	3	4
Stumble	Stumble	3	5

Data are presented as counts (n) of animals showing each class as well as the total number of repetitions.

We matched the observed classes with those described preciously by Wathan et al. (2015), to ensure the consistency off the behavioral assessment [22]. Side and front treadmill bars, individually adjusted to cover the shoulder of the horse, were used as reference lines to ascertain whether abnormal body movements were presented. The front bar was used to identify back offs and discern between mild (back off) and severe (back off II) breaks, which may indicate hesitation or less motivation to trot forward [23]. Side bars were used as reference lines to ascertain head movements (up, down, and lateral movements). Specific non-vocal sounds (snorting), and facial movements, such as mouth opened, dilated nostrils and ears position were carefully examined in a second round of video analysis, wherein only facial movements and sounds were observed.

From the inventory of behaviors exhibited, the classes were tabulated, with their respective descriptions, and the number of all animals that showed each class, as well as the number of repetitions. Then, the evaluation was established for the presence of certain behavior for all animal, accounting for the intervention and time. The code “0” was agreed to express the absence of the behavioral class, and “1” to its presence. All samples of video images were anonymized, to ensure blindness during the inspection of the samples. The observer was a graduated researcher certified EquiFacs coder (https://animalfacs.com/equifacs_new). As our safety protocol does not recommend keeping the horse completely stopped in the treadmill, we only collected images for behavioral assessment during the warm-up and intervention steps. Because introducing a new stimulus into the treadmill room may threat horse’s safety, we also annotated whether they presented severe avoidance behavior, including rearing up, subtle breaks, jumps over the treadmill lateral bars and falls.

### Statistical analysis

Quantitative data was first described (mean ± standard-deviation) for each intervention (VS and Co) and time (baseline, 10’and 12‘), while qualitative data was expressed according to the presence (binary outcome) or summarized as contingency tables. Quantitative dependent variables were analyzed through repeated-measures ANOVA, according to the model y_ijk_ = μ+α_i_+β_j_+(αβ)_ij_+S_k(j)_+ϵ_ijk_ where μ is the overall mean, α_*i*_ is the effect of the *i*th level of the time factor (*i* = before, 10’, 12’), β_*j*_ is the effect of the *j*th level of the interventional factor (*j* = VS, Co) and (αβ)_*ij*_ is the *ij* interaction effect. *S*_*k*(*j*)_ is the random effect of the *k*th animal (*k* = 1, …, *7*), S_k(j)_~ (0,σ^2^_s_) and ϵ_*ijk*_ is the random error assumed ϵ_ijk_~ (0,σ^2^). Once an effect was identified (P< .05), post-hoc analysis was employed (Bonferroni’s correction). Along with descriptive statistics, we expressed the effect size in terms of the difference between marginal means at the 12^th^ minute, i.e. VS minus control, and sun of squares (ges ‐ Generalized Eta Square). Differences between groups at the baseline were assessed through a paired t-test with 95% confidence interval. The association between qualitative dependent variables and the intervention were evaluated using Fisher’s exact test.

## Results

All horses (7/7) were exposed to a warm-up period from the start to the 10^th^ minute of the standardized exercise tests, followed by the intervention (visual stimuli or control) from the 10^th^ to 12^th^ minutes of the SET. Physiological and behavioral markers obtained at the 12^th^ minute, i.e. following visual stimulation/control were considered the primary outcome of this study. There was no baseline difference between groups on the mean heart rate (t = .93; P = .39) blood lactate (t = -.52; P = .62) and glucose (t = -1.63; P = .15), as well as head-neck angle (t = 1.12; P = .30).

There was no effect of group (F = 1.71; P = .25) or time (F = .43; P = .66), but significant group*time interaction for heart rate model (F = 8.88; P < .01). Lactate model analysis revealed significant group*time interaction (F = 8.36; P = .01), and time effect (F = 4.94; P = .04), but no group effect (F = 3.62; P = .13). Glucose model only revealed significant effect of time (F = 6.16; P = .02), while a group*time interaction effect was identified for head-neck angle model (F = 10.45; P < .001).

The analysis of the primary outcomes reveal that that following the visual stimulus, horses had higher heart rate (130.8 ± 27.8 b.p.m) when compared with control stimulus (84.7 ± 15.1 b.p.m) (F = 12.3; P = .017; ges = .56). They also had higher blood lactate values (5.28 ± 1.48 mg/dl) than control (3.27 ± 1.24 mg/dl) (F = 8.65; P = .042; ges = .42). Blood glucose levels of both control and visual stimulus were not statistically different (F = 0.91; P = .43). Head and neck angle increased after visual stimulation (36.43 ± 3.69°) when compared with control (25.14 ± 4.88°) (F = 24.6; P = .003; ges = .69) (Table 2).

**Table 2:** Heart rate (HR), blood lactate and glucose levels as well as head-neck angle of horses engaging a standardized exercise test in a treadmill with (Visual) and without (Control) social facilitation of trotting.

			Before		10’		12’		Dif	95% CI	P-value*
	Group	n	Mean	SD	Mean	SD	Mean	SD			
HR (b.p.m)	Control	7	92.28	40.44	132.16	35.47	84.66	15.06	-46.16	-80.00 to -12.32	.017
HR (b.p.m)	Visual	7	98.42	43.87	104.50	18.26	130.83	27.81			
lactate (mg/dl)	Control	7	4.95	1.98	3.77	1.52	3.271	1.24	-2.22	-4.31 to -0.12	.042
lactate (mg/dl)	Visual	7	5.40	2.49	4.05	1.48	5.28	1.47			
glucose (mg/dl)	Control	7	75,00	4.35	72.71	5.34	70.71	6.21	1.40	-6.57 to 9.37	.650
glucose (mg/dl)	Visual	7	80.71	6.89	75	9.03	68.80	10.94			
Head-neck angle	Control	7	20.28	5.28	24.71	4.85	25.14	4.88	-11.28	-16.8 to -5.71	.003
Head-neck angle	Visual	7	17.14	7.17	22.85	6.66	36.42	3.69			

Data are presented as mean and standard deviation (SD). Difference (Dif) between marginal means of control and visual groups at the 12^th^ minute (Control minus Visual) with 95% confidence interval of the mean difference and P-value of pairwise comparison (Bonferroni’s method).

**Fig 4:**
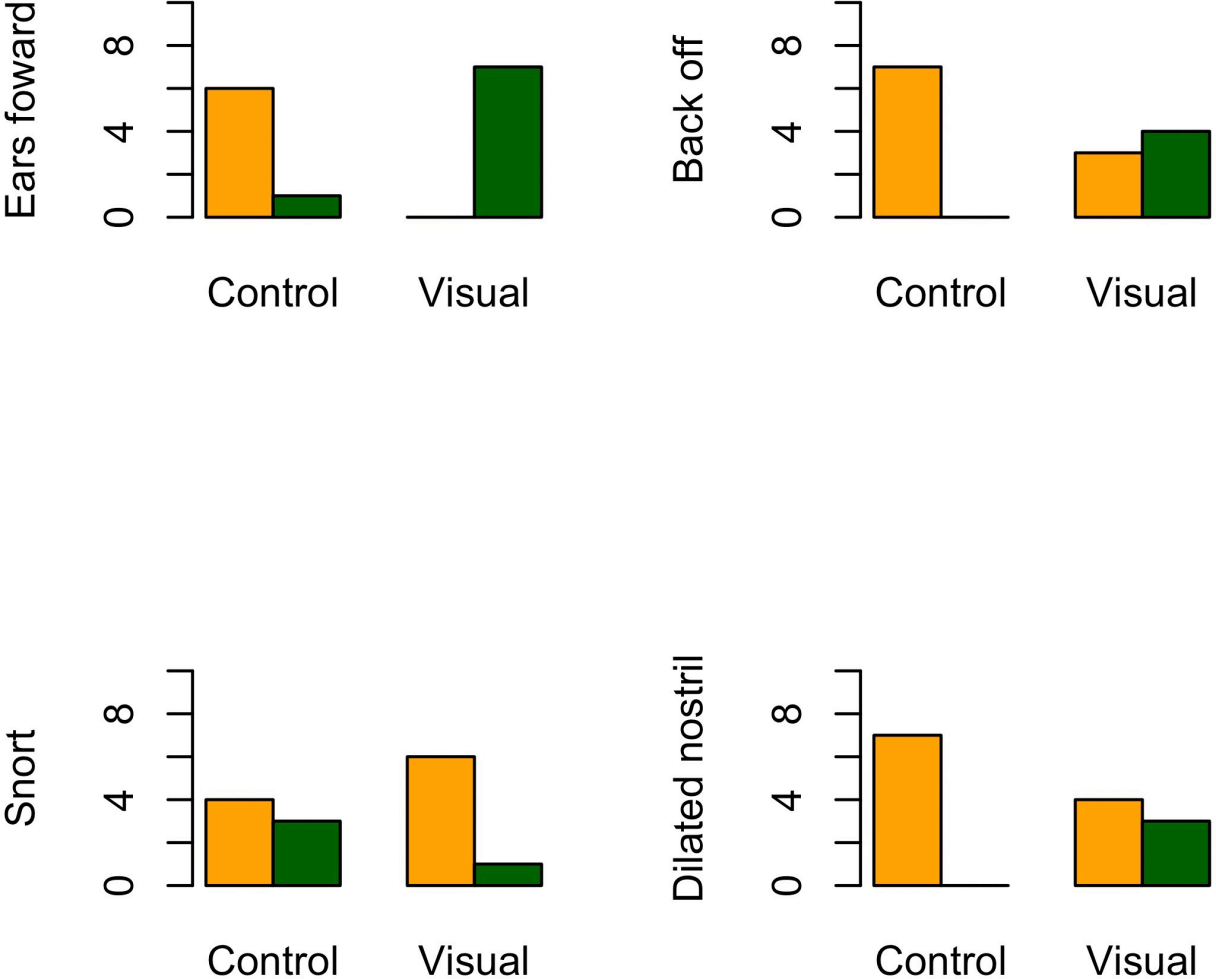
Frequency of behaviors presented during visual stimulation (Visual) and control (Control) in horses undergoing a constant load exercise test on a treadmill. The green bars demonstrate the frequency of horses showing the specific behavior from the 10^th^ to the 12^th^ minutes of the standardized exercise test, while the yellow bars show the frequency of horses not showing the specific behavior.

Because there was no severe avoidance behavior, the intervention was deemed safe and well tolerated. Video image analysis revealed that horses were more likely to present “ears forward” behavior following video stimulation (100% - 7/7) than control (14% - 1/7) (P = .004). There was no association between the presence of “back off” (P = .069), “snorting” (P = .559), or “nostril dilatation” (P = .192), with the exposure to visual stimulation (Fig 4).

Exposure to video images did not influence the prevalence of other classes, including facial and body behaviors in comparison with control during the intervention but did change the prevalence of back offs during the warm-up (P = .029) (Table 3). Horses were more likely to show back offs during the warm-up in the control group than in the visual group.

**Table 3:** Number and frequency of each behavioral class observed during the warm-up and intervention steps of a standardized exercise test on a treadmill, wherein only the projector light was turned on (warm-up) and video images of another horse trotting in front of them (Intervention, Visual) or only a racetrack picture (Intervention, Control) were projected to assess social facilitation of trotting in horses. P-value returns the probability under the null hypothesis that there was no association between the behavioral class and the group (intervention).

Class	Warm-up					Intervention				
	Control		Visual		P	Control		Visual		P
	Number	Frequency	Number	Frequency	value	Number	Frequency	Number	Frequency	value
	n	%	n	%		n	%	n	%	
Back off	6	86	1	14	.029	0/7	0	4/7	57	.069
Mouth opened	3	43	1	14	.559	0/7	0	1/7	14	1.00
Snorting	5	71	7	100	.461	3/7	43	1/7	14	.559
Nostrils opened	1	14	1	14	1.00	0/7	0	3/7	43	.192
Ears forward	2	28	0	0	.461	1/7	14	7/7	100	.004
Side looking	1	14	3	43	.559	1/7	14	3/7	43	.559
Back off II	0/7	0	0/7	0	1.00	0/7	0	3/7	43	.192

Data are presented as absolute (n) and relative (%) frequencies.

## Discussion

We hypothesized that horses are able to respond to social facilitation of trotting, increasing the intensity of their movements to follow or overtake the horse projected in front of them. To verify it, we designed a repeated-measures crossover trial, wherein horses were both exposed to video images of another horse exercising ahead and control image of an empty track. The main finding of this study is that visual stimulus (visual) yielded an overall increase in exercise intensity, as blood lactate and heart rate showed significant increase from control stimulus (Co). Blood lactate is delivered to the vessels as anaerobic lactic pathway starts to yield extra ATP for muscle activity [24]. Recruiting anaerobic lactic paths likely signals an increased demand for energy in locomotory muscle, heart, and brain [25]. The visual stimulus, a non-violent stimulus to trot forward, was deemed to be safe and well tolerated, as no avoidance behavior was observed and no significant increase of either “back offs” or “ears backward” were observed during visual stimulation. Similar frequency of snorts, a non-vocal sound associated with positive emotions [26], across Visual and Co groups both reaffirms the safety of the visual stimulus and the efficacy of the familiarization protocol.

Our findings suggest that horses were more vigilant when exposed to a visual stimulus, as they were more likely to present “ears forward” behavior compared to control group (Co). Horses increase their vigilance by orienting the head, eye, and ears intently towards the object of interest, while preparing the body for a flight or fight response [27]. Forward ear positions are commonly observed during feeding, calm observation, or during positive affiliative interactions with conspecifics or human [28], but may also emerge when horses are attentive to a distant situation [29]. Because ears forward was not mostly linked with agonistic or avoidance behaviors, i.e. horses did not show concurrent vigilant and agonistic behavior, and the fact that “ears forward” occurred together with an increase in exercise intensity and increased head and neck angle (extended neck with raised nose), we may assume that visual stimulus has encouraged the experimental horses to move forward and follow the guide projected ahead, with concurrent demand for cognitive capacities, including visual attention. Although unmeasured in this study, attention is deemed to underly the quality of the decision process in several social contexts [30] and also might be linked with social facilitation of movement in horses.

In addition, horses were more likely to extend the neck and raise the nose following visual stimulation. This behavior has been associated with the need to use the binocular field vision in order to examine distant, frightening objects [31]. Binocular field vision is also essential for stereopsis, i.e. the ability to perceive depth and 3-dimensional structures [32]. Although associated with respiratory dysfunctions [33], riding techniques resulting in the nasal planum held behind the vertical (>90°) remains popular [34]. Recent survey suggested that cervical hyperflexion may impair horses’ vision during exercise [35], but was not confirmed by other survey indicating that horses are able to move the ocular globe to compensate abnormal head position [36]. Despite being an unformal observation, saccadic i.e. ocular movements were observed in horses under visual stimulation in this study. We observed that horses sustained more pronounced eye movement when exposed to visual stimuli than control. Because this observation was made informally, i.e. not systematically computed over all video frames, it was not presented in the results. Increased saccadic movements were likely an attempt to improve the visualization of the images and may reveal uncertainty over its content but suggest that horses can use multiple sensory paths during a complex sensorial task. Finally, these findings also suggest that driver should allow the horse to move its head sustaining normal visual behavior during exercise, to adapt to the incoming environment and setting appropriate movements to deal with it.

There is evidence that attention is related to the quality of the decision-making process in contexts such as predator detection, competition, communication, and social learning [37,38]. In our study, the increased vigilance state likely encouraged general biomechanical adjustments, wherein a preparatory or anticipatory states are prioritized [38]. Because there was a front bar in the treadmill, horses were not able to trot forward. On the other hand, we kept the lead rope long to allow them to move backward, change stride pattern (increase or decrease stride length, duration, or frequency), and redistribute the weight, assuming collected postures. A novel methodology to enable the self regulation of treadmill speed may benefits future studies on social facilitation of movement in horses.

Despite the promising findings reported here, we highlight some limitations of this study: (1) our SET did not allow horses to freely regulate trotting speed during visual stimulation, therefore it’s unclear whether they were more or less likely to accelerate toward the projected video and (2) the study design did not allow for a double blinding the experiments, as researchers conducting the exercise test should be knew whether horses were engaging VS or control. Conversely, video images were anonymized before its analysis.

According to the authors’ knowledge this is the first study that investigated the effect of a visual stimulus designed to promote social facilitation of movement on horses’ behavior and physiology. Future studies may develop methods to quantify the reaction time, as well as the basis of the decision-making process for movement initiation/acceleration. Further comparisons between visual stimulus and whip use effects on horse’s willingness to move faster are also plausible. Understanding horses’ preference to work collectively, as well as inter-individual variability in responding to visual stimulus should be essential for improving the welfare of horses performing advanced training. Possible associations between reactivity and performance in competitions that involve quick decisions or precision in changes of direction can be tested. Traditional equestrian disciplines such as cross-country, polo, and dressage likely require high level of perception-action coordination.

## Conclusion

We observed minimum risk of exposing horses to visual stimulus during treadmill exercise. The visual stimulus, designed to promote social facilitation of trotting, increased horse’s exercise intensity and shifted the behavior to an increased vigilance. Our findings validate the use of social facilitation to encourage horses to move forward, on a treadmill, avoiding the use of the whip. We also highlight the need for future studies on innovative riding techniques, which allows the expression of normal visual behavior during exercise.

## Supporting information

S1 Fig Racetrack image utilised as control visual stimulus. (TIF)

S1 File “Formal analysis”: Formal analysis, demonstrating summary statistics, model’s building and output. (HTML)

S1 Data Dataset. (XLSX)
