# Supplementary figures and images for: Social facilitation of trotting: Can horses perceive and adapt to the movement of another horse?
Source: PLoS One. 2024 Aug 26;19(8):e0309474. doi: 10.1371/journal.pone.0309474 (PMC11346917; doi:10.1371/journal.pone.0309474)

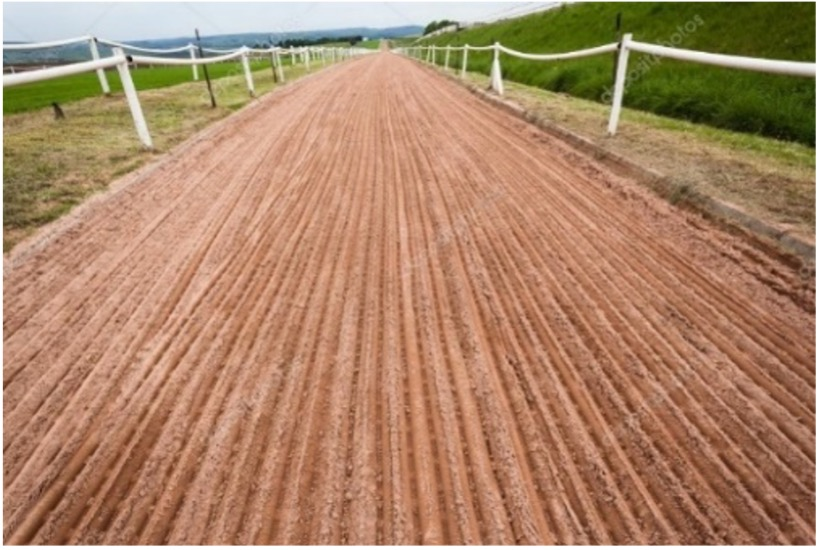

Supplement: S1 Fig — (TIF) [file pone.0309474.s001.tif]
