# Supplementary material for: Social facilitation of trotting: Can horses perceive and adapt to the movement of another horse?
Source: PLoS One. 2024 Aug 26;19(8):e0309474. doi: 10.1371/journal.pone.0309474 (PMC11346917; doi:10.1371/journal.pone.0309474)
Supplement: S1 File — (HTML) [file pone.0309474.s002.html]

Social facilitation of trotting in horses


Code 

- Show All Code
- Hide All Code
- Download Rmd

# Social facilitation of trotting in horses

Packages


```
library(readxl)
library(rstatix)
library(rstatix)
library(reshape)
library(tidyverse)
library(dplyr)
library(ggpubr)
library(datarium)
library(sciplot)
library(plyr)
library(naniar)
```


Data importation


```
setwd("/Users/Paulo/Desktop/PESQUISA/1 Working Papers/Visual /1 Dados")
data<-read_xlsx("Dados_Paulo.xlsx", sheet="matriz", col_names = T)
str(data)
```


```
tibble [42 × 16] (S3: tbl_df/tbl/data.frame)
 $ animal                   : chr [1:42] "pampa" "pampa" "pampa" "pampa" ...
 $ sequencia                : chr [1:42] "tc" "tc" "tc" "tc" ...
 $ periodo                  : num [1:42] 2 2 2 1 1 1 1 1 1 2 ...
 $ grupo                    : chr [1:42] "CONTROLE" "CONTROLE" "CONTROLE" "TESTE" ...
 $ tempo                    : num [1:42] 0 1 2 0 1 2 0 1 2 0 ...
 $ fc                       : num [1:42] 26 NA NA 25 NA NA 108 90 76 112 ...
 $ lactato                  : num [1:42] 6.4 3.9 2.9 10.1 7.2 7.4 8.3 5.8 3.9 5.2 ...
 $ glicose                  : num [1:42] 81 82 80 86 82 83 70 73 74 85 ...
 $ atrasar                  : num [1:42] NA 1 0 NA 0 0 NA 1 0 NA ...
 $ abrir_a_boca             : num [1:42] NA 0 0 NA 0 0 NA 0 0 NA ...
 $ bufar                    : num [1:42] NA 0 0 NA 1 1 NA 1 1 NA ...
 $ dilatar_narinas          : num [1:42] NA 0 0 NA 0 0 NA 0 0 NA ...
 $ fixar_orelhas_para_frente: num [1:42] NA 0 0 NA 0 1 NA 1 0 NA ...
 $ olhar_para_lado          : num [1:42] NA 0 0 NA 0 0 NA 1 1 NA ...
 $ recuar                   : num [1:42] NA 0 0 NA 0 0 NA 0 0 NA ...
 $ angulo_da_cabeca         : num [1:42] 27 32 35 31 32 36 13 20 22 14 ...
```


```
vis_miss(data)
```


Set up and transform variables


```
data$tempo<-as.factor(data$tempo)
data$grupo<- as.factor(data$grupo)
data$animal<- as.factor(data$animal)
data$periodo<- as.factor(data$periodo)
```


Descriptive statistics


```
tab<- data %>%
  group_by(tempo, grupo) %>%
  get_summary_stats(c(fc, lactato, glicose, angulo_da_cabeca ), type = "mean_sd")
print(format.data.frame(tab, digits = 8))
```


Data preparation: complete case analysis


```
data.fc<- na.omit(data[,1:6])
data.lac<- na.omit(data[,c(1,2,3,4,5,7)])
data.glic<- na.omit(data[,c(1,2,3,4,5,8)])
data.ang<- na.pass(data[,c(1,2,3,4,5,16)])
data.lac$loglac<- log(data.lac$lactato)
```


Assessing baseline differences


```
bas_fc<-t.test(fc~grupo, data = subset(data.fc, tempo==0), paired=T)
bas_la<-t.test(lactato~grupo, data = subset(data.lac, tempo==0), paired=T)
bas_gli<-t.test(glicose~grupo, data = subset(data.glic, tempo==0), paired=T)
bas_ang<-t.test(angulo_da_cabeca ~grupo, data = subset(data.ang, tempo==0), paired=T)
print(bas_fc)
```


```
    Paired t-test

data:  fc by grupo
t = -0.93244, df = 6, p-value = 0.3871
alternative hypothesis: true mean difference is not equal to 0
95 percent confidence interval:
 -22.263041   9.977327
sample estimates:
mean difference 
      -6.142857
```


```
print(bas_la)
```


```
    Paired t-test

data:  lactato by grupo
t = -0.52332, df = 6, p-value = 0.6195
alternative hypothesis: true mean difference is not equal to 0
95 percent confidence interval:
 -2.513532  1.627818
sample estimates:
mean difference 
     -0.4428571
```


```
print(bas_gli)
```


```
    Paired t-test

data:  glicose by grupo
t = -1.628, df = 6, p-value = 0.1546
alternative hypothesis: true mean difference is not equal to 0
95 percent confidence interval:
 -14.302817   2.874245
sample estimates:
mean difference 
      -5.714286
```


```
print(bas_ang)
```


```
    Paired t-test

data:  angulo_da_cabeca by grupo
t = 1.1232, df = 6, p-value = 0.3043
alternative hypothesis: true mean difference is not equal to 0
95 percent confidence interval:
 -3.704095  9.989810
sample estimates:
mean difference 
       3.142857
```


Building 2-way ANOVA-RM models


```
fc.aov <- anova_test(data = data.fc, dv = fc, wid = animal,
  within = c(grupo, tempo))
la.aov <- anova_test(data = data.lac, dv = loglac, wid = animal,
  within = c(grupo, tempo))
gli.aov <- anova_test(data = data.glic, dv = glicose, wid = animal,
  within = c(grupo, tempo))
ang.aov <- anova_test(data = data.ang, dv = angulo_da_cabeca , wid = animal,
  within = c(grupo, tempo))
```


Printing FC Model


```
get_anova_table(fc.aov)
```


```
ANOVA Table (type III tests)

       Effect DFn DFd     F     p p<.05   ges
1       grupo   1   5 1.719 0.247       0.028
2       tempo   2  10 0.431 0.661       0.040
3 grupo:tempo   2  10 8.881 0.006     * 0.263
```


Printing Lactate Model


```
get_anova_table(la.aov)
```


```
ANOVA Table (type III tests)

       Effect DFn DFd     F     p p<.05   ges
1       grupo   1   4 3.621 0.130       0.144
2       tempo   2   8 4.941 0.040     * 0.067
3 grupo:tempo   2   8 8.361 0.011     * 0.070
```


Printing Glucose Model


```
get_anova_table(gli.aov)
```


```
ANOVA Table (type III tests)

       Effect DFn DFd     F     p p<.05   ges
1       grupo   1   4 0.047 0.840       0.002
2       tempo   2   8 6.162 0.024     * 0.201
3 grupo:tempo   2   8 0.918 0.438       0.015
```


Printing Head-neck Model


```
get_anova_table(ang.aov)
```


```
ANOVA Table (type III tests)

       Effect DFn DFd      F        p p<.05   ges
1       grupo   1   6  5.398 5.90e-02       0.040
2       tempo   2  12 38.349 6.13e-06     * 0.481
3 grupo:tempo   2  12 10.488 2.00e-03     * 0.287
```


Pairwise comparison


```
pc.fc <- data.fc %>%group_by(tempo) %>%anova_test(dv = fc, wid = animal, within = grupo) %>%
  get_anova_table() %>%
  adjust_pvalue(method = "bonferroni")
pc.lq <- data.lac %>%group_by(tempo) %>%anova_test(dv = loglac, wid = animal, within = grupo) %>%get_anova_table() %>%
  adjust_pvalue(method = "bonferroni")
pc.gli <- data.glic %>%group_by(tempo) %>%anova_test(dv = glicose, wid = animal, within = grupo) %>%get_anova_table() %>%
  adjust_pvalue(method = "bonferroni")
pc.ang <- data.ang %>%group_by(tempo) %>%anova_test(dv = angulo_da_cabeca, wid = animal, within = grupo) %>%get_anova_table() %>% adjust_pvalue(method = "bonferroni")

pc.fc
```


```
pc.lq
```


```
pc.gli
```


```
pc.ang
```


Inspecting residuals of ANOVA models


```
resi.fc <- residuals(fc.model$Within)
resi.la <- residuals(la.model$Within)
resi.gli <- residuals(gli.model$Within)
resi.ang <- residuals(ang.model$Within)
shapiro.test(resi.fc)
```


```
    Shapiro-Wilk normality test

data:  resi.fc
W = 0.95484, p-value = 0.4756
```


```
shapiro.test(resi.la)
```


```
    Shapiro-Wilk normality test

data:  resi.la
W = 0.97422, p-value = 0.8564
```


```
shapiro.test(resi.gli)
```


```
    Shapiro-Wilk normality test

data:  resi.gli
W = 0.93851, p-value = 0.2479
```


```
shapiro.test(resi.ang)
```


```
    Shapiro-Wilk normality test

data:  resi.ang
W = 0.99377, p-value = 0.9999
```


#Behavior variable setup


```
data$abrir_a_boca<-as.factor(data$abrir_a_boca)
data$atrasar<-as.factor(data$atrasar)
data$bufar<-as.factor(data$bufar)
data$dilatar_narinas<-as.factor(data$dilatar_narinas)
data$fixar_orelhas_para_frente<-as.factor(data$fixar_orelhas_para_frente)
data$olhar_para_lado<-as.factor(data$olhar_para_lado)
data$recuar<-as.factor(data$recuar)
data.beh<-na.omit(data[,c(4,5,9:15)])
str(data.beh)
```


```
tibble [28 × 9] (S3: tbl_df/tbl/data.frame)
 $ grupo                    : Factor w/ 2 levels "CONTROLE","TESTE": 1 1 2 2 1 1 2 2 1 1 ...
 $ tempo                    : Factor w/ 3 levels "0","1","2": 2 3 2 3 2 3 2 3 2 3 ...
 $ atrasar                  : Factor w/ 2 levels "0","1": 2 1 1 1 2 1 2 1 2 1 ...
 $ abrir_a_boca             : Factor w/ 2 levels "0","1": 1 1 1 1 1 1 1 1 2 1 ...
 $ bufar                    : Factor w/ 2 levels "0","1": 1 1 2 2 2 2 2 1 2 1 ...
 $ dilatar_narinas          : Factor w/ 2 levels "0","1": 1 1 1 1 1 1 1 1 1 1 ...
 $ fixar_orelhas_para_frente: Factor w/ 2 levels "0","1": 1 1 1 2 2 1 1 2 1 1 ...
 $ olhar_para_lado          : Factor w/ 2 levels "0","1": 1 1 1 1 2 2 2 1 1 1 ...
 $ recuar                   : Factor w/ 2 levels "0","1": 1 1 1 1 1 1 1 2 1 1 ...
 - attr(*, "na.action")= 'omit' Named int [1:14] 1 4 7 10 13 16 19 22 25 28 ...
  ..- attr(*, "names")= chr [1:14] "1" "4" "7" "10" ...
```


Intervention: Testing the association between each class and the
independent group variable


```
# Função para realizar o teste qui-quadrado
test_chi_square <- function(data, var, group) {
  # Crie uma tabela de contingência
  tbl <- table(data[[var]], data[[group]])
  
  # Realize o teste qui-quadrado
  test <- fisher.test(tbl)
  
  # Retorne o resultado do teste
  return(test)
}

variaveis<- colnames(data.beh[2:8])
print(variaveis)
```


```
[1] "tempo"                     "atrasar"                   "abrir_a_boca"             
[4] "bufar"                     "dilatar_narinas"           "fixar_orelhas_para_frente"
[7] "olhar_para_lado"
```


```
# Realize o teste para cada variável qualitativa
resultados <- lapply(variaveis, function(var) test_chi_square(subset(data, tempo==2), var, "grupo"))

# Exiba os resultados
names(resultados) <- variaveis
resultados
```


```
$tempo

    Fisher's Exact Test for Count Data

data:  tbl
p-value = 1
alternative hypothesis: two.sided


$atrasar

    Fisher's Exact Test for Count Data

data:  tbl
p-value = 0.06993
alternative hypothesis: true odds ratio is not equal to 1
95 percent confidence interval:
 0.8498871       Inf
sample estimates:
odds ratio 
       Inf 


$abrir_a_boca

    Fisher's Exact Test for Count Data

data:  tbl
p-value = 1
alternative hypothesis: true odds ratio is not equal to 1
95 percent confidence interval:
 0.02564066        Inf
sample estimates:
odds ratio 
       Inf 


$bufar

    Fisher's Exact Test for Count Data

data:  tbl
p-value = 0.5594
alternative hypothesis: true odds ratio is not equal to 1
95 percent confidence interval:
 0.003646918 4.442542966
sample estimates:
odds ratio 
 0.2480182 


$dilatar_narinas

    Fisher's Exact Test for Count Data

data:  tbl
p-value = 0.1923
alternative hypothesis: true odds ratio is not equal to 1
95 percent confidence interval:
 0.4614829       Inf
sample estimates:
odds ratio 
       Inf 


$fixar_orelhas_para_frente

    Fisher's Exact Test for Count Data

data:  tbl
p-value = 0.004662
alternative hypothesis: true odds ratio is not equal to 1
95 percent confidence interval:
 2.508783      Inf
sample estimates:
odds ratio 
       Inf 


$olhar_para_lado

    Fisher's Exact Test for Count Data

data:  tbl
p-value = 0.5594
alternative hypothesis: true odds ratio is not equal to 1
95 percent confidence interval:
   0.2250963 274.2041246
sample estimates:
odds ratio 
  4.031951
```


Warm-up: Testing the association between each class and the
independent group variable


```
# Realize o teste para cada variável qualitativa
resultados <- lapply(variaveis, function(var) test_chi_square(subset(data.beh, tempo==1), var, "grupo"))

# Exiba os resultados
names(resultados) <- variaveis
resultados
```


```
$tempo

    Fisher's Exact Test for Count Data

data:  tbl
p-value = 1
alternative hypothesis: two.sided


$atrasar

    Fisher's Exact Test for Count Data

data:  tbl
p-value = 0.02914
alternative hypothesis: true odds ratio is not equal to 1
95 percent confidence interval:
 0.000531383 0.799817637
sample estimates:
odds ratio 
0.04256025 


$abrir_a_boca

    Fisher's Exact Test for Count Data

data:  tbl
p-value = 0.5594
alternative hypothesis: true odds ratio is not equal to 1
95 percent confidence interval:
 0.003646918 4.442542966
sample estimates:
odds ratio 
 0.2480182 


$bufar

    Fisher's Exact Test for Count Data

data:  tbl
p-value = 0.4615
alternative hypothesis: true odds ratio is not equal to 1
95 percent confidence interval:
 0.1928147       Inf
sample estimates:
odds ratio 
       Inf 


$dilatar_narinas

    Fisher's Exact Test for Count Data

data:  tbl
p-value = 1
alternative hypothesis: true odds ratio is not equal to 1
95 percent confidence interval:
  0.01094678 91.35109674
sample estimates:
odds ratio 
         1 


$fixar_orelhas_para_frente

    Fisher's Exact Test for Count Data

data:  tbl
p-value = 0.4615
alternative hypothesis: true odds ratio is not equal to 1
95 percent confidence interval:
 0.000000 5.186326
sample estimates:
odds ratio 
         0 


$olhar_para_lado

    Fisher's Exact Test for Count Data

data:  tbl
p-value = 0.5594
alternative hypothesis: true odds ratio is not equal to 1
95 percent confidence interval:
   0.2250963 274.2041246
sample estimates:
odds ratio 
  4.031951
```


Power analysis Assuming a level of sphericity equat to 0.1, an effect
size F equal to 8.36 and alpha equal to 0.05.


```
pw.fc<-wp.rmanova(n = 7, ng = 2, nm = 3, f = 8.88, nscor = .1,
  alpha = 0.05, power = NULL, type = 2)
print(pw.fc)
```


```
Repeated-measures ANOVA analysis

    n    f ng nm nscor alpha     power
    7 8.88  2  3   0.1  0.05 0.9584677

NOTE: Power analysis for interaction-effect test
URL: http://psychstat.org/rmanova
```


```
pw.la<-wp.rmanova(n = 7, ng = 2, nm = 3, f = 8.36, nscor = .1,
  alpha = 0.05, power = NULL, type = 2)
print(pw.la)
```


```
Repeated-measures ANOVA analysis

    n    f ng nm nscor alpha     power
    7 8.36  2  3   0.1  0.05 0.9447488

NOTE: Power analysis for interaction-effect test
URL: http://psychstat.org/rmanova
```


```
pw.gli<-wp.rmanova(n = 7, ng = 2, nm = 3, f = 0.91, nscor = .1,
  alpha = 0.05, power = NULL, type = 2)
print(pw.gli)
```


```
Repeated-measures ANOVA analysis

    n    f ng nm nscor alpha     power
    7 0.91  2  3   0.1  0.05 0.1154752

NOTE: Power analysis for interaction-effect test
URL: http://psychstat.org/rmanova
```


```
pw.ang<-wp.rmanova(n = 7, ng = 2, nm = 3, f = 10.49, nscor = .1,
  alpha = 0.05, power = NULL, type = 2)
print(pw.ang)
```


```
Repeated-measures ANOVA analysis

    n     f ng nm nscor alpha     power
    7 10.49  2  3   0.1  0.05 0.9841791

NOTE: Power analysis for interaction-effect test
URL: http://psychstat.org/rmanova
```


LS0tCnRpdGxlOiAiU29jaWFsIGZhY2lsaXRhdGlvbiBvZiB0cm90dGluZyBpbiBob3JzZXMiCm91dHB1dDoKICBodG1sX2RvY3VtZW50OgogICAgZGZfcHJpbnQ6IHBhZ2VkCiAgaHRtbF9ub3RlYm9vazogZGVmYXVsdAogIHBkZl9kb2N1bWVudDogZGVmYXVsdAotLS0KUGFja2FnZXMKYGBge3J9CmxpYnJhcnkocmVhZHhsKQpsaWJyYXJ5KHJzdGF0aXgpCmxpYnJhcnkocnN0YXRpeCkKbGlicmFyeShyZXNoYXBlKQpsaWJyYXJ5KHRpZHl2ZXJzZSkKbGlicmFyeShkcGx5cikKbGlicmFyeShnZ3B1YnIpCmxpYnJhcnkoZGF0YXJpdW0pCmxpYnJhcnkoc2NpcGxvdCkKbGlicmFyeShwbHlyKQpsaWJyYXJ5KG5hbmlhcikKbGlicmFyeShXZWJQb3dlcikKYGBgCgpEYXRhIGltcG9ydGF0aW9uCmBgYHtyfQpzZXR3ZCgiL1VzZXJzL1BhdWxvL0Rlc2t0b3AvUEVTUVVJU0EvMSBXb3JraW5nIFBhcGVycy9WaXN1YWwgLzEgRGFkb3MiKQpkYXRhPC1yZWFkX3hsc3goIkRhZG9zX1BhdWxvLnhsc3giLCBzaGVldD0ibWF0cml6IiwgY29sX25hbWVzID0gVCkKc3RyKGRhdGEpCnZpc19taXNzKGRhdGEpCmBgYAoKU2V0IHVwIGFuZCB0cmFuc2Zvcm0gdmFyaWFibGVzCmBgYHtyfQpkYXRhJHRlbXBvPC1hcy5mYWN0b3IoZGF0YSR0ZW1wbykKZGF0YSRncnVwbzwtIGFzLmZhY3RvcihkYXRhJGdydXBvKQpkYXRhJGFuaW1hbDwtIGFzLmZhY3RvcihkYXRhJGFuaW1hbCkKZGF0YSRwZXJpb2RvPC0gYXMuZmFjdG9yKGRhdGEkcGVyaW9kbykKYGBgCgpEZXNjcmlwdGl2ZSBzdGF0aXN0aWNzCmBgYHtyfQp0YWI8LSBkYXRhICU+JQogIGdyb3VwX2J5KHRlbXBvLCBncnVwbykgJT4lCiAgZ2V0X3N1bW1hcnlfc3RhdHMoYyhmYywgbGFjdGF0bywgZ2xpY29zZSwgYW5ndWxvX2RhX2NhYmVjYSApLCB0eXBlID0gIm1lYW5fc2QiKQpwcmludChmb3JtYXQuZGF0YS5mcmFtZSh0YWIsIGRpZ2l0cyA9IDgpKQpgYGAKRGF0YSBwcmVwYXJhdGlvbjogY29tcGxldGUgY2FzZSBhbmFseXNpcwpgYGB7cn0KZGF0YS5mYzwtIG5hLm9taXQoZGF0YVssMTo2XSkKZGF0YS5sYWM8LSBuYS5vbWl0KGRhdGFbLGMoMSwyLDMsNCw1LDcpXSkKZGF0YS5nbGljPC0gbmEub21pdChkYXRhWyxjKDEsMiwzLDQsNSw4KV0pCmRhdGEuYW5nPC0gbmEucGFzcyhkYXRhWyxjKDEsMiwzLDQsNSwxNildKQpkYXRhLmxhYyRsb2dsYWM8LSBsb2coZGF0YS5sYWMkbGFjdGF0bykKYGBgCgpBc3Nlc3NpbmcgYmFzZWxpbmUgZGlmZmVyZW5jZXMKYGBge3J9CmJhc19mYzwtdC50ZXN0KGZjfmdydXBvLCBkYXRhID0gc3Vic2V0KGRhdGEuZmMsIHRlbXBvPT0wKSwgcGFpcmVkPVQpCmJhc19sYTwtdC50ZXN0KGxhY3RhdG9+Z3J1cG8sIGRhdGEgPSBzdWJzZXQoZGF0YS5sYWMsIHRlbXBvPT0wKSwgcGFpcmVkPVQpCmJhc19nbGk8LXQudGVzdChnbGljb3NlfmdydXBvLCBkYXRhID0gc3Vic2V0KGRhdGEuZ2xpYywgdGVtcG89PTApLCBwYWlyZWQ9VCkKYmFzX2FuZzwtdC50ZXN0KGFuZ3Vsb19kYV9jYWJlY2EgfmdydXBvLCBkYXRhID0gc3Vic2V0KGRhdGEuYW5nLCB0ZW1wbz09MCksIHBhaXJlZD1UKQpwcmludChiYXNfZmMpCnByaW50KGJhc19sYSkKcHJpbnQoYmFzX2dsaSkKcHJpbnQoYmFzX2FuZykKYGBgCgoKQnVpbGRpbmcgMi13YXkgQU5PVkEtUk0gbW9kZWxzCmBgYHtyfQpmYy5hb3YgPC0gYW5vdmFfdGVzdChkYXRhID0gZGF0YS5mYywgZHYgPSBmYywgd2lkID0gYW5pbWFsLAogIHdpdGhpbiA9IGMoZ3J1cG8sIHRlbXBvKSkKbGEuYW92IDwtIGFub3ZhX3Rlc3QoZGF0YSA9IGRhdGEubGFjLCBkdiA9IGxvZ2xhYywgd2lkID0gYW5pbWFsLAogIHdpdGhpbiA9IGMoZ3J1cG8sIHRlbXBvKSkKZ2xpLmFvdiA8LSBhbm92YV90ZXN0KGRhdGEgPSBkYXRhLmdsaWMsIGR2ID0gZ2xpY29zZSwgd2lkID0gYW5pbWFsLAogIHdpdGhpbiA9IGMoZ3J1cG8sIHRlbXBvKSkKYW5nLmFvdiA8LSBhbm92YV90ZXN0KGRhdGEgPSBkYXRhLmFuZywgZHYgPSBhbmd1bG9fZGFfY2FiZWNhICwgd2lkID0gYW5pbWFsLAogIHdpdGhpbiA9IGMoZ3J1cG8sIHRlbXBvKSkKYGBgCgpQcmludGluZyBGQyBNb2RlbApgYGB7cn0KZ2V0X2Fub3ZhX3RhYmxlKGZjLmFvdikKYGBgClByaW50aW5nIExhY3RhdGUgTW9kZWwKYGBge3J9CmdldF9hbm92YV90YWJsZShsYS5hb3YpCmBgYApQcmludGluZyBHbHVjb3NlIE1vZGVsCmBgYHtyfQpnZXRfYW5vdmFfdGFibGUoZ2xpLmFvdikKYGBgClByaW50aW5nIEhlYWQtbmVjayBNb2RlbApgYGB7cn0KZ2V0X2Fub3ZhX3RhYmxlKGFuZy5hb3YpCmBgYAoKUGFpcndpc2UgY29tcGFyaXNvbgpgYGB7cn0KcGMuZmMgPC0gZGF0YS5mYyAlPiVncm91cF9ieSh0ZW1wbykgJT4lYW5vdmFfdGVzdChkdiA9IGZjLCB3aWQgPSBhbmltYWwsIHdpdGhpbiA9IGdydXBvKSAlPiUKICBnZXRfYW5vdmFfdGFibGUoKSAlPiUKICBhZGp1c3RfcHZhbHVlKG1ldGhvZCA9ICJib25mZXJyb25pIikKcGMubHEgPC0gZGF0YS5sYWMgJT4lZ3JvdXBfYnkodGVtcG8pICU+JWFub3ZhX3Rlc3QoZHYgPSBsb2dsYWMsIHdpZCA9IGFuaW1hbCwgd2l0aGluID0gZ3J1cG8pICU+JWdldF9hbm92YV90YWJsZSgpICU+JQogIGFkanVzdF9wdmFsdWUobWV0aG9kID0gImJvbmZlcnJvbmkiKQpwYy5nbGkgPC0gZGF0YS5nbGljICU+JWdyb3VwX2J5KHRlbXBvKSAlPiVhbm92YV90ZXN0KGR2ID0gZ2xpY29zZSwgd2lkID0gYW5pbWFsLCB3aXRoaW4gPSBncnVwbykgJT4lZ2V0X2Fub3ZhX3RhYmxlKCkgJT4lCiAgYWRqdXN0X3B2YWx1ZShtZXRob2QgPSAiYm9uZmVycm9uaSIpCnBjLmFuZyA8LSBkYXRhLmFuZyAlPiVncm91cF9ieSh0ZW1wbykgJT4lYW5vdmFfdGVzdChkdiA9IGFuZ3Vsb19kYV9jYWJlY2EsIHdpZCA9IGFuaW1hbCwgd2l0aGluID0gZ3J1cG8pICU+JWdldF9hbm92YV90YWJsZSgpICU+JSBhZGp1c3RfcHZhbHVlKG1ldGhvZCA9ICJib25mZXJyb25pIikKCnBjLmZjCnBjLmxxCnBjLmdsaQpwYy5hbmcKYGBgCgpJbnNwZWN0aW5nIHJlc2lkdWFscyBvZiBBTk9WQSBtb2RlbHMKYGBge3J9CnJlc2kuZmMgPC0gcmVzaWR1YWxzKGZjLm1vZGVsJFdpdGhpbikKcmVzaS5sYSA8LSByZXNpZHVhbHMobGEubW9kZWwkV2l0aGluKQpyZXNpLmdsaSA8LSByZXNpZHVhbHMoZ2xpLm1vZGVsJFdpdGhpbikKcmVzaS5hbmcgPC0gcmVzaWR1YWxzKGFuZy5tb2RlbCRXaXRoaW4pCnNoYXBpcm8udGVzdChyZXNpLmZjKQpzaGFwaXJvLnRlc3QocmVzaS5sYSkKc2hhcGlyby50ZXN0KHJlc2kuZ2xpKQpzaGFwaXJvLnRlc3QocmVzaS5hbmcpCmBgYAojQmVoYXZpb3IKdmFyaWFibGUgc2V0dXAKYGBge3J9CmRhdGEkYWJyaXJfYV9ib2NhPC1hcy5mYWN0b3IoZGF0YSRhYnJpcl9hX2JvY2EpCmRhdGEkYXRyYXNhcjwtYXMuZmFjdG9yKGRhdGEkYXRyYXNhcikKZGF0YSRidWZhcjwtYXMuZmFjdG9yKGRhdGEkYnVmYXIpCmRhdGEkZGlsYXRhcl9uYXJpbmFzPC1hcy5mYWN0b3IoZGF0YSRkaWxhdGFyX25hcmluYXMpCmRhdGEkZml4YXJfb3JlbGhhc19wYXJhX2ZyZW50ZTwtYXMuZmFjdG9yKGRhdGEkZml4YXJfb3JlbGhhc19wYXJhX2ZyZW50ZSkKZGF0YSRvbGhhcl9wYXJhX2xhZG88LWFzLmZhY3RvcihkYXRhJG9saGFyX3BhcmFfbGFkbykKZGF0YSRyZWN1YXI8LWFzLmZhY3RvcihkYXRhJHJlY3VhcikKZGF0YS5iZWg8LW5hLm9taXQoZGF0YVssYyg0LDUsOToxNSldKQpzdHIoZGF0YS5iZWgpCmBgYAoKSW50ZXJ2ZW50aW9uOgpUZXN0aW5nIHRoZSBhc3NvY2lhdGlvbiBiZXR3ZWVuIGVhY2ggY2xhc3MgYW5kIHRoZSBpbmRlcGVuZGVudCBncm91cCB2YXJpYWJsZQpgYGB7cn0KIyBGdW7Dp8OjbyBwYXJhIHJlYWxpemFyIG8gdGVzdGUgcXVpLXF1YWRyYWRvCnRlc3RfY2hpX3NxdWFyZSA8LSBmdW5jdGlvbihkYXRhLCB2YXIsIGdyb3VwKSB7CiAgIyBDcmllIHVtYSB0YWJlbGEgZGUgY29udGluZ8OqbmNpYQogIHRibCA8LSB0YWJsZShkYXRhW1t2YXJdXSwgZGF0YVtbZ3JvdXBdXSkKICAKICAjIFJlYWxpemUgbyB0ZXN0ZSBxdWktcXVhZHJhZG8KICB0ZXN0IDwtIGZpc2hlci50ZXN0KHRibCkKICAKICAjIFJldG9ybmUgbyByZXN1bHRhZG8gZG8gdGVzdGUKICByZXR1cm4odGVzdCkKfQoKdmFyaWF2ZWlzPC0gY29sbmFtZXMoZGF0YS5iZWhbMjo4XSkKcHJpbnQodmFyaWF2ZWlzKQoKIyBSZWFsaXplIG8gdGVzdGUgcGFyYSBjYWRhIHZhcmnDoXZlbCBxdWFsaXRhdGl2YQpyZXN1bHRhZG9zIDwtIGxhcHBseSh2YXJpYXZlaXMsIGZ1bmN0aW9uKHZhcikgdGVzdF9jaGlfc3F1YXJlKHN1YnNldChkYXRhLCB0ZW1wbz09MiksIHZhciwgImdydXBvIikpCgojIEV4aWJhIG9zIHJlc3VsdGFkb3MKbmFtZXMocmVzdWx0YWRvcykgPC0gdmFyaWF2ZWlzCnJlc3VsdGFkb3MKCmBgYApXYXJtLXVwOgpUZXN0aW5nIHRoZSBhc3NvY2lhdGlvbiBiZXR3ZWVuIGVhY2ggY2xhc3MgYW5kIHRoZSBpbmRlcGVuZGVudCBncm91cCB2YXJpYWJsZQpgYGB7cn0KIyBSZWFsaXplIG8gdGVzdGUgcGFyYSBjYWRhIHZhcmnDoXZlbCBxdWFsaXRhdGl2YQpyZXN1bHRhZG9zIDwtIGxhcHBseSh2YXJpYXZlaXMsIGZ1bmN0aW9uKHZhcikgdGVzdF9jaGlfc3F1YXJlKHN1YnNldChkYXRhLmJlaCwgdGVtcG89PTEpLCB2YXIsICJncnVwbyIpKQoKIyBFeGliYSBvcyByZXN1bHRhZG9zCm5hbWVzKHJlc3VsdGFkb3MpIDwtIHZhcmlhdmVpcwpyZXN1bHRhZG9zCgpgYGAKClBvd2VyIGFuYWx5c2lzCkFzc3VtaW5nIGEgbGV2ZWwgb2Ygc3BoZXJpY2l0eSBlcXVhdCB0byAwLjEsIGFuIGVmZmVjdCBzaXplIEYgZXF1YWwgdG8gOC4zNiBhbmQgYWxwaGEgZXF1YWwgdG8gMC4wNS4KYGBge3J9CnB3LmZjPC13cC5ybWFub3ZhKG4gPSA3LCBuZyA9IDIsIG5tID0gMywgZiA9IDguODgsIG5zY29yID0gLjEsCiAgYWxwaGEgPSAwLjA1LCBwb3dlciA9IE5VTEwsIHR5cGUgPSAyKQpwcmludChwdy5mYykKcHcubGE8LXdwLnJtYW5vdmEobiA9IDcsIG5nID0gMiwgbm0gPSAzLCBmID0gOC4zNiwgbnNjb3IgPSAuMSwKICBhbHBoYSA9IDAuMDUsIHBvd2VyID0gTlVMTCwgdHlwZSA9IDIpCnByaW50KHB3LmxhKQpwdy5nbGk8LXdwLnJtYW5vdmEobiA9IDcsIG5nID0gMiwgbm0gPSAzLCBmID0gMC45MSwgbnNjb3IgPSAuMSwKICBhbHBoYSA9IDAuMDUsIHBvd2VyID0gTlVMTCwgdHlwZSA9IDIpCnByaW50KHB3LmdsaSkKcHcuYW5nPC13cC5ybWFub3ZhKG4gPSA3LCBuZyA9IDIsIG5tID0gMywgZiA9IDEwLjQ5LCBuc2NvciA9IC4xLAogIGFscGhhID0gMC4wNSwgcG93ZXIgPSBOVUxMLCB0eXBlID0gMikKcHJpbnQocHcuYW5nKQpgYGAKCg==
